# *In Vivo* Bypass of 8-oxodG

**DOI:** 10.1371/journal.pgen.1003682

**Published:** 2013-08-01

**Authors:** Gina P. Rodriguez, Joseph B. Song, Gray F. Crouse

**Affiliations:** 1 Department of Biology, Emory University, Atlanta, Georgia, United States of America; 2 Winship Cancer Institute, Emory University, Atlanta, Georgia, United States of America; Duke University, United States of America

## Abstract

8-oxoG is one of the most common and mutagenic DNA base lesions caused by oxidative damage. However, it has not been possible to study the replication of a known 8-oxoG base *in vivo* in order to determine the accuracy of its replication, the influence of various components on that accuracy, and the extent to which an 8-oxoG might present a barrier to replication. We have been able to place a single 8-oxoG into the *Saccharomyces cerevisiae* chromosome in a defined location using single-strand oligonucleotide transformation and to study its replication in a fully normal chromosome context. During replication, 8-oxoG is recognized as a lesion and triggers a switch to translesion synthesis by Pol η, which replicates 8-oxoG with an accuracy (insertion of a C opposite the 8-oxoG) of approximately 94%. In the absence of Pol η, template switching to the newly synthesized sister chromatid is observed at least one third of the time; replication of the 8-oxoG in the absence of Pol η is less than 40% accurate. The mismatch repair (MMR) system plays an important role in 8-oxoG replication. Template switching is blocked by MMR and replication accuracy even in the absence of Pol η is approximately 95% when MMR is active. These findings indicate that in light of the overlapping mechanisms by which errors in 8-oxoG replication can be avoided in the cell, the mutagenic threat of 8-oxoG is due more to its abundance than the effect of a single lesion. In addition, the methods used here should be applicable to the study of any lesion that can be stably incorporated into synthetic oligonucleotides.

## Author Summary

In the course of normal cellular functions, many types of reactive oxygen species are produced that can lead to oxidative damage in the cell. DNA bases are subject to the formation of various oxidative lesions; one of the most common is the production of 8-oxoG which can pair relatively well with A instead of C, leading to GC→TA transversion mutations. In this work, we have been able to place a single 8-oxoG in the yeast chromosome and observe its replication. We find that in a wild-type cell its replication is surprisingly accurate due primarily to two components: DNA mismatch repair, which recognizes an A inserted opposite the 8-oxoG and initiates its removal and subsequent re-replication; and translesion synthesis in which the existence of the 8-oxoG induces a switch from a normal replicating DNA polymerase to a specialized DNA polymerase, Pol η, that can accurately replicate an 8-oxoG. We also find that the 8-oxoG can cause the replicating strand to shift to the newly replicated strand in the sister chromatid, thus avoiding the 8-oxoG lesion. These findings indicate not only how cells deal with a known DNA lesion, but also demonstrate how other such lesions can be studied in the future.

## Introduction

All DNA bases are subject to a variety of different types of damage due to reactive oxygen species (ROS) [1]. Among the most common and most mutagenic is 7,8-dihydro-8-oxoguanine, or 8-oxoG, which is mutagenic because of its tendency to pair with adenine and thus create GC to TA transversion mutations [2], [3]. In the yeast *Saccharomyces cerevisiae* there are several mechanisms either to repair 8-oxoG lesions or to prevent 8-oxoG-induced mutations. 8-oxoG lesions opposite C, which would be formed by oxidative damage of double-stranded DNA, are removed by the glycosylase Ogg1 [4], [5], which has little if any activity on 8-oxoG paired with other bases [6]. Mismatch repair (MMR) plays an important role in preventing mutations due to oxidative damage [7], and it has been shown that yeast MutSα, consisting of the Msh2 and Msh6 subunits, recognizes A replicated opposite an 8-oxoG lesion and thereby prevents mutations [8]. Thus in *S. cerevisiae*, MMR appears to replace the function of MutY, which is absent [8], [9]. MutSβ, consisting of the Msh2 and Msh3 subunits appears to play no role in 8-oxoG repair [8].

For 8-oxoG lesions that are not removed prior to replication, translesion DNA synthesis (TLS) is importantly involved in bypass, with Pol η playing the major role in yeast. A variety of biochemical experiments using oligonucleotide templates with an 8-oxoG lesion have demonstrated that Pol η replicates through an 8-oxoG lesion, usually inserting a C [10]–[12]. This accuracy is explained by structural studies that show Pol η with a template containing an 8-oxoG lesion can hold the lesion in an *anti* conformation, permitting a C to be inserted [13]. In contrast, Pol δ is ten-fold less accurate and efficient in bypassing 8-oxoG [14] and Pol ε does not bypass 8-oxoG at normal dNTP concentrations, but does, inaccurately, at damage-induced levels of dNTPs [15]. Genetic studies are more complicated because the existence of an 8-oxoG lesion can be inferred only by its mutation signature, generally in an *ogg1* background that greatly increases the amount of 8-oxoG in DNA. Such studies were used to show the involvement of MMR in preventing mutations due to 8-oxoG [8], the role of Pol η in accurate replication of 8-oxoG [11], [16], and the lack of a significant role for Pol ζ [16], [17]. The interplay of TLS and MMR is not completely clear. It was proposed that MMR was responsible for recruiting Pol η for bypass [18] but a detailed study of 8-oxoG bypass and repair concluded that Pol η acted independently of MMR [19]. It appears that monoubiquitination of PCNA is necessary for most TLS and in yeast this step is carried out by the Rad6-Rad18 heterodimer [20], [21]. Genetic studies implicate *RAD6* and *RAD18* as well as *RAD30* (the gene encoding Pol η) but not *REV3* (the gene encoding the catalytic subunit of Pol ζ) in 8-oxoG tolerance [16].

Most TLS is assumed to occur at the replication fork [20], [21], although it can occur after S phase [22]. Furthermore, as there appear to be different replicative polymerases on the leading and lagging strands of replication [23], one might expect 8-oxoG tolerance could exhibit strand differences. There are only a limited number of such studies. Using a reversion analysis of a *URA3* mutation, it was found that 8-oxoG was preferentially repaired on the lagging strand of replication [24]; most of the differential repair was ascribed to the preferential activity of MMR on the lagging strand [25]. Using a mutation analysis of *ogg1*-dependent mutations in a *SUP4-o* reporter assay, the lagging strand bias of MutSα was observed, as well as a lagging strand bias for accurate Pol η bypass [19].

It is not clear what effect an 8-oxoG lesion has on replication. Some work has suggested that an 8-oxoG lesion has no effect on replication [10], [18], whereas a stall site was observed *in vitro* at a nucleotide prior to an 8-oxoG lesion with Pol δ but not Pol η [11]. An *in vivo* study inferred replication stalling or blockage from a mutational analysis [19]. Lesions that block or stall replication forks appear to be tolerated, especially in yeast, by homologous recombination [26]. Recent interest has focused on tolerance mechanisms by template switching in which a blocked 3′ end invades the replicating sister strand, either by a fork regression or strand invasion [26]. Such mechanisms of template switching appear to be dependent on polyubiquitination of PCNA by a complex of Ubc13-Mms2-Rad5 [20]. Because the substrate of Ubc13-Mms2-Rad5 is PCNA monoubiquitinated by Rad6-Rad18, template switching would also be expected to be dependent on Rad6 and Rad18 [20]. In addition to its role in polyubiquitination, the helicase function of Rad5 may also be important in template switching [27], [28].

Rather than using an *ogg1* mutant background, a more direct method of analyzing 8-oxoG bypass *in vivo* would be to introduce DNA containing a defined lesion directly into cells. Plasmids containing a single-strand gap with an 8-oxoG or 8-oxoG in duplex DNA have been introduced into *E. coli* and mammalian cells [29]–[32] and a plasmid treated with methylene blue to induce oxidative damage was introduced into yeast for analysis [33]. The problem with the use of plasmids for analysis, in addition to the difficulty of substrate construction, is that the mechanism of replication may differ from that within the chromosome and various forms of recombinational bypass may also differ. Another approach would be to transform cells with single-stranded oligonucleotides (oligos) containing an 8-oxoG lesion. Transformation of yeast with oligos was first performed in Fred Sherman's laboratory [34], [35] and the method has subsequently been used to study various lesions carried into yeast by oligos [36]–[41]. However, in most cases the lesion itself was responsible for generating a phenotype and with one exception [36] the mechanism of transformation with oligos was not fully understood.

In order to study 8-oxoG bypass, we wanted to introduce the 8-oxoG on an oligo that would create a selectable phenotype that would be independent of the presence of the 8-oxoG lesion. Such an experimental design allows us to study both replication across the 8-oxoG and bypass of the 8-oxoG by template switching outside of a context of overall increased oxidative damage in the cell. As detailed below, we find evidence that the 8-oxoG lesion does stall replication; that only Pol η is able to replicate 8-oxoG accurately; that template switching is invoked frequently in the absence of Pol η; and that MMR strongly influences the outcome of 8-oxoG replication.

## Results

**Figure 1:**
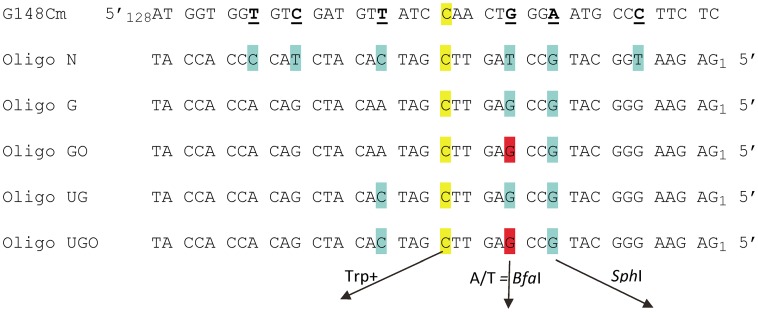
Sequences of *TRP5* mutant regions and oligonucleotides used for reversion analysis. The *trp5*-*G148Cm* mutant contains several changes designed to create additional completely degenerate third codon positions, with those of interest underlined; the sequence shown here begins at nt 128. The C at position 148 that must change to G in order to restore a Trp+ phenotype is highlighted in yellow. Oligo N creates 7 mismatches (highlighted in yellow and blue) upon annealing with the G148Cm sequence; oligos are numbered from the 5′ end. Oligo G and UG create a subset of those mismatches as indicated. Oligos GO and UGO are identical to Oligos G and UG except that the base highlighted in red is an 8-oxoG. If A is inserted opposite the 8-oxoG during replication, a novel *Bfa*I restriction site is created; similarly, the G highlighted in blue, if incorporated, creates a novel *Sph*I restriction site.

We sought a system in which a damaged base could be placed into the chromosome independent of an oligo-induced reversion event and so needed a low spontaneous reversion rate coupled with a tight selection. We turned to the set of *trp5* point mutations we previously constructed [42]. These strains contain a mutation at either nucleotide position 148 or 149 and can only be reverted to the wild type phenotype by restoring the original *TRP5* sequence [42]. The plan was therefore to revert a mutant *TRP5* gene with an oligonucleotide containing the wild-type base along with a damaged base at a different location in the oligo. A potential problem was that the region surrounding the mutant base is highly conserved, constraining the location of any damaged base. We therefore created the mutant *trp5-G148Cm* gene (Figure 1) [43]. Because this mutant *trp5-G148Cm* gene is placed close to a dependable origin of replication, and is present in both orientations relative to the origin, we know which strand is replicated as leading and which as lagging and can reverse the replication strands by using a strain of opposite *TRP5* orientation [42]. In order to use oligos to incorporate a segment of DNA, it was necessary to know the frequency of co-incorporation of nucleotides in a given oligo. Using oligos with markers spread throughout the length of the oligo (Oligo N, Figure 1), we determined that for an oligo of 40 nt in length, a central core of 10–15 nt was incorporated with a greater than 90% frequency [43]. Those results suggested that it was feasible to use oligos of that length for our experiments.

### An assay system for 8-oxodG bypass

We had initially hoped to investigate damaged base bypass by transforming with an oligo which contained one normal base to revert the Trp- phenotype and another damaged base placed in a silent position where any base incorporation would be tolerated. However, our prior experiments [43] as well as a number of preliminary experiments indicated that we needed a method to mark incorporation of bases on both sides of the damaged base in order to be sure that we were observing bypass, and not partial incorporation of the relevant region of the oligo. These goals were accomplished by transforming with Oligos G and GO (Figure 1). The C at position 20, highlighted in yellow, creates a Trp+ phenotype upon incorporation; the G at position 12, highlighted in blue, if incorporated, creates a new *Sph*I site. The G at position 15 is an 8-oxodG in Oligo GO and is highlighted in red, forming an 8-oxoG-G mismatch with the *trp5-G148Cm* sequence. Oligo G is identical, with a G instead of an 8-oxodG at position 15. This mismatch was deliberately chosen, as one of the main glycosylases processing 8-oxodG, Ogg1, should have little or no activity on an 8-oxodG-G mismatch [44], [45], and the efficiency of its removal by another glycosylase, Ntg1, is low, if it exists [46]. In addition, 8-oxodG, when bypassed, is very unlikely to template a G, so if the original sequence at that position is maintained, that would be strong evidence either of removal of the 8-oxodG before replication, or a failure to bypass the 8-oxodG. The expectation for 8-oxodG bypass is that either a C or A is incorporated. If an A is incorporated opposite the 8-oxodG, a *Bfa*I site is created, thus allowing a simple restriction digestion to indicate a mutagenic bypass of the 8-oxodG. In summary, at the site in question, a G on the coding strand indicates that 8-oxoG was either not used as a template for replication or was removed before replication, a C indicates that 8-oxoG was bypassed accurately, and an A indicates inaccurate replication of 8-oxoG.

**Figure 2:**
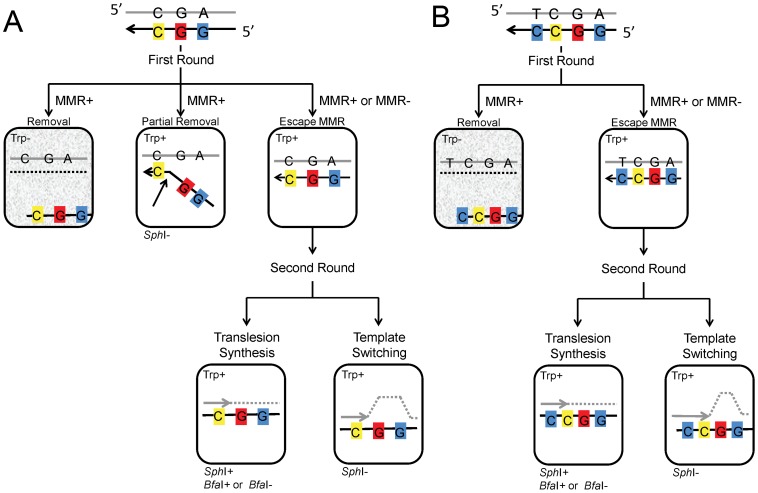
Models for incorporation of oligo GO and UGO. (A) Incorporation of Oligo GO creates three mismatches; if the oligo is not removed before the second round, replication of the C, indicated in yellow, would result in a Trp+ phenotype, replication of the 8-oxoG, indicated in red, would create a *Bfa*I site if an A were incorporated, and replication of the G indicated in blue would create an *Sph*I site. In the first round, usually the entire portion of the oligo containing the marked bases remains or is removed by MMR. However, when MMR is present, the segment of the oligo containing the 5′-two mismatches can be removed, but the segment creating the Trp+ phenotype can be left, presumably due to failure of MMR to recognized the C-C mismatch (see text). If the marked segment of Oligo GO persists to the second round of replication, translesion synthesis creates an *Sph*I site and a *Bfa*I site if the 8-oxoG is bypassed with an A. If the 8-oxoG induces template switching, no *Sph*I site is created. (B) Incorporation of Oligo UGO creates an additional mismatch with the C indicated in blue, 3′ of the C-C mismatch. Due to this additional mismatch, in the first round, either the entire oligo remains, or is removed. The results of the second round of replication are identical to that of Oligo GO in (A). Cells with a gray background are Trp- and thus do not survive selection.

The overall design of the assay system and its expected results are illustrated in Figure 2A. Incorporation of the oligo can be selected by the Trp+ phenotype, and given that only 7 nt separate the base creating the Trp+ phenotype and the base creating an *Sph*I site, we initially expected that all Trp+ cells should contain a new *Sph*I site. What we found as analysis proceeded is that a fraction of oligos, even those containing all normal bases, exhibited “partial removal” as indicated in Figure 2A: in the presence of MMR, a substantial fraction of cells (as much as 30% or more) transformed by Oligo G (containing only normal bases) were Trp+ but did not contain an *Sph*I site [43]. Those results were explained by a failure of MMR to recognize the C-C mismatch created by the oligo during MMR-directed excision from the 5′ end of the oligo [43]. Such results were seen only in the presence of MMR and with Oligo G and Oligo GO, but not with Oligo UG or UGO, as will be detailed below. It is in the second round in which the oligo sequence, now fully incorporated into the genome, is replicated for the first time. In Trp+ cells that were transformed by Oligo GO and contain the *Sph*I site, DNA synthesis must have used the 8-oxoG for a template, and the base inserted can be subsequently analyzed. In the absence of MMR, all Trp+ cells would be expected to contain an *Sph*I site, and that is true for cells transformed by Oligo G but not for all strains transformed by Oligo GO. The failure of cells transformed by Oligo GO to contain an *Sph*I site could be explained by the process of template switching, in which the replication fork switches to use the newly replicated strand of the sister chromatid [26].

### 8-oxodG induces template switching in the absence of Pol η

**Figure 3:**
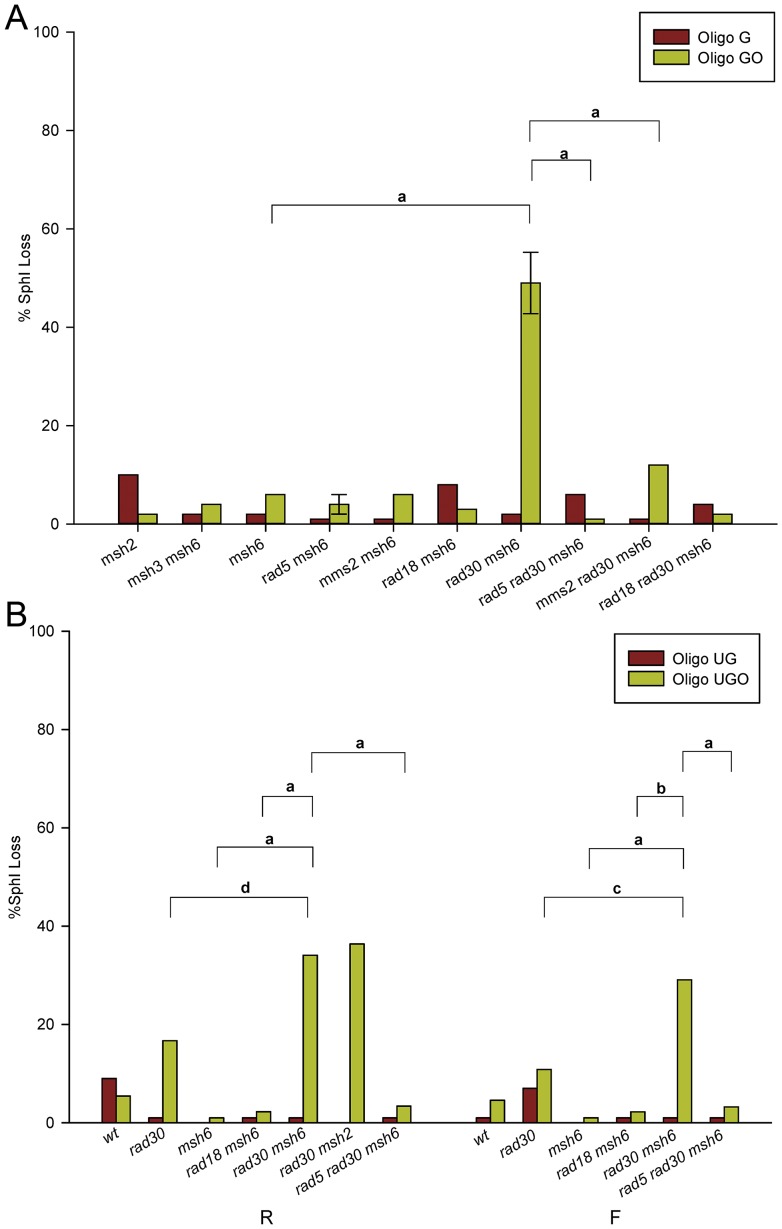
Template switching induced by 8-oxoG. Template switching was determined as those transformants that did not contain the *Sph*I restriction site introduced by the oligo. More than 40 Trp+ revertants were assayed for the presence of an *Sph*I site in each strain of the indicated genotype. Error bars represent standard deviation of the mean for cases in which three or more independent experiments were done. The 8-oxoG lesion is replicated in the second round; replication would be on the leading strand in R strains. Horizontal bars indicate selected genotype comparisons, with the letter above the bars indicating the probability of the null hypothesis that the results of transformation with Oligo GO or Oligo UGO in the two strains are the same. For **a**: P<0.0001; **b**: P = 0.0002; **c**: P = 0.004; **d**: P = 0.02. (A) Strains in the R orientation transformed with Oligo G or Oligo GO. (B) Experiments as in (A) using Oligo UG or Oligo UGO in strains of both orientations.

Strains with a variety of different genotypes were transformed by Oligo G and Oligo GO and assayed for the presence of an *Sph*I site. The results for strains of the R orientation are shown in Figure 3A. Results for strains of the F orientation are shown in Figure S1 and the numbers of colonies analyzed for each strain are given in Table S1. Because of the problem of partial oligo removal discussed above, strains with an active MMR cannot be analyzed for template switching (i.e. Trp+ transformants lacking an *Sph*I site) with Oligo GO. In MMR-defective strains, with the exception of *rad30 msh6* strains lacking both MMR and Pol η, the *Sph*I site is created in almost all Oligo GO transformants. If the lack of the *Sph*I site in that background is due to template switching, it should be blocked by loss of Rad5, Mms2, or Rad18 [20], [27], [47], [48]. That is seen to be true, as *rad5 rad30 msh6*, *mms2 rad30 msh6*, and *rad18 rad30 msh6* strains show minimal loss of the *Sph*I site.

Our previous results had suggested that placing a base creating an additional mismatch 3′ of the C-C mismatch in Oligo G would prevent the partial removal of the oligo illustrated in Figure 2A [43]. Therefore, we used Oligo UG and Oligo UGO (Figure 1) to repeat a subset of the experiments shown in Figures 3A and S1. The results assaying presence of the *Sph*I site in both R and F strains are presented in Figure 3B. As observed with Oligo GO, Oligo UGO displays template switching in the absence of both MMR and Pol η (Figure 3B). Loss of the *Sph*I site is suppressed in *rad5 rad30 msh6* strains. Oligo UG transformants in the presence of MMR showed little loss of the *Sph*I site (Figure 3B). Oligo UGO transformants in *rad30* strains also demonstrated little *Sph*I site loss, and were significantly reduced in template switching compared to *rad30 msh6* strains (Figure 3B). Therefore we can conclude that most template switching is suppressed by MMR. It appears in Figure 3B that the level of *Sph*I site loss in *rad30* Oligo UGO transformants is somewhat elevated compared to wild-type strains. The difference is not statistically significant in strains with the F orientation, and is only marginally significant (P = 0.03) in the R orientation.

### Accuracy of 8-oxoG bypass depends on MMR and Pol η

**Figure 4:**
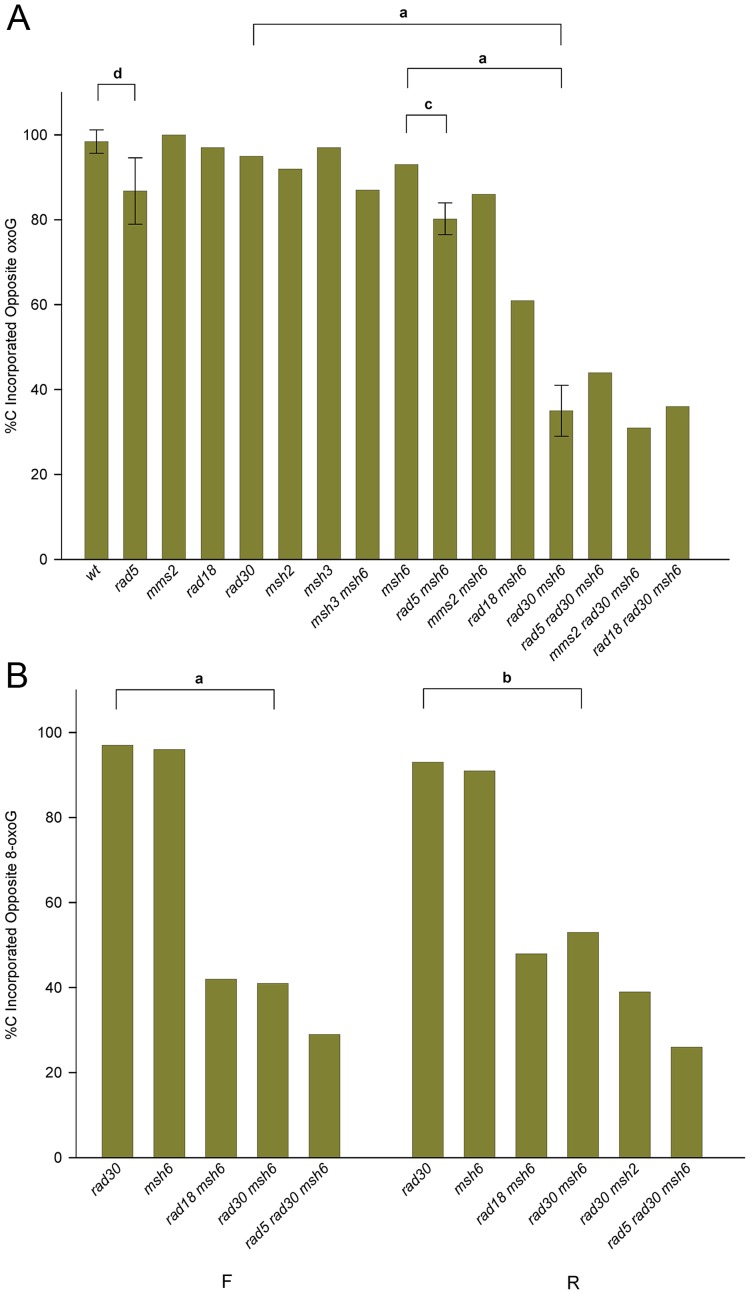
Accuracy of 8-oxoG replication. Trp+ revertants containing an *Sph*I site were assayed for the presence of a *Bfa*I site resulting from insertion of an A opposite 8-oxoG in strains of the indicated genotype transformed with an 8-oxoG-containing oligo; those transformants lacking the *Bfa*I site had a C inserted opposite the 8-oxoG. In most cases, more than 40 *Sph*I containing revertants were assayed. Note that the 8-oxoG lesion is replicated in the second round; thus for example replication is on the leading strand in R strains. The error bars and horizontal bars are as in Figure 3. For **a**: P<0.0001; **b**: P = 0.0005; **c**: P = 0.01; **d**: P = 0.04. (A) Strains in the R orientation transformed with Oligo GO. (B) Experiments as in (A) using Oligo UGO instead of Oligo GO in strains of both orientations.

8-oxodG is considered to be extremely mutagenic due to the frequency of misreplication, with an A inserted opposite the 8-oxodG. In order to measure the bypass accuracy of the introduced 8-oxodG, we selected only those revertants that were both Trp+ and contained an *Sph*I site, as all of those revertants should have incorporated the intervening 8-oxodG into the genome. As illustrated in Figure 2, the 8-oxodG in oligos GO and UGO was placed opposite a G in the genome; thus removal of the 8-oxoG lesion would have resulted in retention of the original sequence at that point. Replication of the 8-oxoG lesion would be expected to yield only a C for accurate bypass or an A for inaccurate bypass, both leading to a change of sequence at that position. The replication accuracy could have been directly determined by sequencing each one of the revertants. However, as indicated in Figure 1, inaccurate replication with an A creates a novel *Bfa*I site, allowing a direct measurement of accuracy without sequencing. In order to assess the validity of this approach, we sequenced 129 Trp+ revertants that contained the introduced *Sph*I site but lacked the *Bfa*I restriction site and found 126 C, 1 T and 2 G at that position, thus confirming the utility of the restriction site assay and the assumption that a C would be found in such cases; 22 out of 22 sequences that contained the *Bfa*I site had an A as expected. The result of this assay in a variety of genetic backgrounds using Oligo GO is shown for strains of R orientation in Figure 4A and for the F orientation in Figure S2. All numbers are given in Table S1. To our surprise, not only was replication extremely accurate in wild-type cells, it was also highly accurate in the absence of MMR, averaging 94% in MMR-defective strains of both orientations compared to 97% in wild-type strains; the difference is not statistically significant. The source of the accurate replication was clearly Pol η, as in MMR-deficient strains in the absence of Pol η, the accuracy dropped to 36% in R orientation and 44% in F orientation (Figures 4A and S2; Table S1). The resulting 8-oxoG-A mismatch was efficiently recognized and corrected by MMR, as the replication accuracy in *rad30* strains was 92% in R and 93% in F (Figures 4A and S2; Table S1), neither of which was significantly different from that measured in wild type or MMR-defective strains.

In order to confirm our results with Oligo GO, we conducted a reduced set of experiments with Oligo UGO (Figure 4B; Table S1). The accuracies measured in either *rad30* or *msh6* strains were not significantly different from each other. The double mutant combinations of *msh6 rad30* were significantly lower, at 44% in F and 36% in R orientation.

The graphs of accuracy in Figures 4 and S2 demonstrate that there are basically two categories of strains: those strains with deficient Pol η and MMR, and those that have at least one of the two pathways intact (as discussed above, Rad18 is thought to be necessary for Pol η function, which is consistent with our results). In general within each group there is no statistically significant difference among strains, and there is a significant difference between strains in the two groups. It appears that *rad5* strains could be an exception. In both the R (Figure 4A) and F (Figure S2) orientations, accuracy in *rad5* strains is lower than in wild-type, and accuracy in *rad5 msh6* strains is lower than in *msh6* strains. The P values are marginal, ranging from 0.01 to 0.04, but the pattern is consistent in the four comparisons. Another question is whether there are differences between the accuracies observed in the two orientations of the *TRP5* gene. The measured accuracy in R strains is lower than in F strains for Oligo GO in *msh2*, *msh6*, *msh3 msh6*, and for Oligo UGO in *msh6* strains. However, only by combining the results in *msh2*, *msh3*, and *msh3 msh6* strains for Oligo GO does the difference approach statistical significance (P = 0.05). Because the 8-oxoG is replicated in the second round, replication of the 8-oxoG would be on the leading strand in strains with the R orientation.

## Discussion

In this work, we have developed a method of analyzing bypass of 8-oxoG *in vivo* by using oligos to place a single 8-oxoG in a defined location in the chromosome. Once incorporated into the chromosome, the 8-oxoG lesion is not replicated until a second cell cycle, such that the measured replication is of a lesion fully integrated into the chromosome. The replication of 8-oxoG was surprisingly accurate and that accuracy was due to the synergistic action of MMR and Pol η. Although the general expectation was that 8-oxoG would not affect replication, 8-oxoG-induced template switching was observed, but for the most part only in the absence of both MMR and Pol η.

### The accuracy of 8-oxoG bypass

We know that Trp+ revertants containing a new *Sph*I site must have resulted from replication past the 8-oxoG and can therefore measure the accuracy of that bypass. 8-oxoG is considered to be a very mutagenic lesion; therefore it was somewhat of a surprise that in wild-type cells it was replicated quite accurately. The accuracy of replication was 98% in F strains (replication on the lagging strand) and 97% in R strains. Even more surprising was the accuracy in MMR-deficient cells; pooling data from all genotypes lacking MutSα (*msh2*, *msh6*, *msh3 msh6*) gave 96% accuracy in F strains and 91% in R. As noted above, this difference is of marginal statistical significance (P = 0.05), but it is in agreement with experiments that showed a lagging strand bias for Pol η [19]. One important distinction between our measurement and other *in vivo* measurements is that our strains contain one 8-oxoG lesion above the background level of such lesions, whereas most other measurements have been made in Ogg1-deficient strains in which one would expect large numbers of additional 8-oxoG lesions. Given that the amount of MutSα in cells is low (one estimate is 1230 molecules of Msh2p and 5330 molecules of Msh6p per cell [49]), elevated levels of 8-oxoG in the cell could potentially titrate out MutSα. The source of accurate replication of 8-oxoG in MutSα-deficient strains is clearly Pol η, as in cells deleted for Pol η, 8-oxoG is replicated accurately only 40% of the time (Figure 4 and Table S1). Pol η is also in relatively low abundance in yeast (an estimated 1860 molecules per cell [49]) again suggesting a potentially misleading picture of 8-oxoG replication in cells with elevated levels of 8-oxoG. Thus our measurements indicate the levels of accuracy to be expected for repair of spontaneous levels of oxidative damage in normal conditions, but might not apply for cells under oxidative stress or with reduced levels of MMR or Pol η. Given that the accuracy of bypass in the absence of MMR was due to Pol η, our results support the independence of MMR and Pol η in maintenance of accuracy, as previously observed [19], and are not consistent with a model in which MMR is required to recruit Pol η [18].

Previous *in vitro* measurements had determined that yeast Pol η could replicate an 8-oxoG much more accurately than could Pol δ [11], [14], but it is impossible to extrapolate from such *in vitro* experiments using single DNA polymerases to an *in vivo* situation in a chromosomal context with multiple DNA polymerases available. The low accuracy observed in MMR- and Pol η-defective strains (40%) suggests that no other DNA polymerase in the cell is able to replicate 8-oxoG accurately. Therefore the high accuracy of 8-oxoG replication observed in MMR-defective strains indicates that most of the 8-oxoG replication must be due to Pol η. (Suppose Pol η is used for 75% of 8-oxoG bypass, and other polymerases with only 40% accuracy are used the rest of the time; the overall accuracy of bypass in the absence of MMR would be approximately 85%, considerably lower than what we observe.) *In vitro* experiments found a strong stall site with Pol δ just before replication of an 8-oxoG [11]; such a stall is likely the signal responsible on either replication strand for switching to synthesis by Pol η or switching templates.

Particularly because of the different abilities of Pol δ and Pol ε to bypass an 8-oxoG *in vitro* [14], [15], one might have expected a difference in replication fidelity due to replication of leading and lagging strands by different DNA polymerases [23], although we see little evidence for that here. The similarity in accuracy of leading and lagging strands in the absence of Pol η is surprising, particularly given the expected difference in the ability of Pol δ and Pol ε to bypass 8-oxoG. Our data suggest that the 8-oxoG lesion causes a stall; it has been hypothesized that a lesion on the leading strand could induce a switch to continued synthesis on the leading strand by Pol δ [50] and so it is possible that the synthesis observed on either strand across 8-oxoG in the absence of Pol η could be due primarily to Pol δ.

### 8-oxoG-induced template switching

The design of these experiments made it possible to observe template switching, in which the replicating DNA polymerase uses DNA from the replicating sister chromatid as a source of template [26]. In strains deficient in MMR, template switching was observed only in strains lacking Pol η (Rad30), and in those cases, occurred in about half of the replication events on both the leading and lagging strands of replication (Figures 3 and S1). As expected for template switching, these events were not observed in strains lacking Rad5, Mms2, or Rad18.

Template switching was measured as Trp+ revertants that did not contain the *Sph*I site introduced by the oligo. As explained above, experiments with Oligo GO could not examine possible template switching in the presence of MMR because of the loss of part of the oligo during transformation in some Trp+ colonies (Figure 2A). Such partial loss was not observed with Oligo UGO, and those experiments showed that MMR suppresses template switching, as such events were significantly lower in *rad30* strains compared to *rad30 msh6* strains in both orientations (Figure 3B). It is possible that some template switching events were missed in these assays, as only 4 nucleotides separate the 8-oxoG from the C needed to produce Trp+ cells. If in template switching, there is loss of more than 4 bases from the 3′ invading end, the resulting strain would be Trp- and therefore not observed.

Template switching could occur via fork regression on the leading strand [26], but on the lagging strand must occur via a mechanism involving homologous recombination [47]. What triggers template switching and how does MMR suppress template switching? A stall by the replicative DNA polymerase in advance of the 8-oxoG is a strong candidate for a template switching signal. As postulated above, as a replicative DNA polymerase encountered an 8-oxoG, it would stall and either induce a switch to Pol η replication or the replicative polymerase would switch templates and bypass the lesion in an error-free manner. In the presence of MMR, presumably the same template switching would occur but when the DNA copied from the sister chromatid was brought back to pair with the template strand, MMR would recognize the 8-oxoG-G mispair and initiate removal of the newly synthesized DNA, thus abolishing the effect of the template switch.

It is evident from Figures 3A and S1 that deletion of either *RAD5* or *MMS2* blocks template switching. However, deletion of *RAD5* in either wild-type or *msh6* strains appears to somewhat decrease accuracy in Oligo GO transformants, whereas deletion of *MMS2* in the same strains does not (Figures 4A and S2). That result is consistent with a role for Rad5 in addition to template switching [51]. A *rad5* strain is more sensitive to UV damage than an *mms2* strain [52] and a role for Rad5 was observed in TLS of UV damage independent of Mms2-Ubc13 [53]. Although on many substrates the Rad5-dependent TLS might be mutagenic [54], it would appear from our work that the Rad5-mediated events are accurate in replicating 8-oxoG.

The sequence context of a lesion can affect its fate. The *trp5-G148Cm* strains we have made could accommodate a lesion at several different positions, and thus somewhat different sequence contexts. Another option would be to place a lesion in a sequence context of choice and integrate it into a strain that would select for the loop integration [36]. The potential difficulty with such methods is that the spontaneous background of such reversion events, particularly in the absence of MMR, is considerably higher than in the *trp5-G148Cm* strains. The use of oligos to place defined DNA damage at unique places in the chromosome is potentially very informative. With proper markers in the oligos, we have shown that the fate of a defined lesion can be measured in a completely normal chromosome context in a variety of genetic backgrounds.

## Materials and Methods

### Yeast strains

The *trp5-G148Cm* mutation was created as described for the other *trp5* mutations [42] using *delitto perfetto* [55] and created the sequence C GATGTTATCCAACTGGGA starting at position 138 of *TRP5* with mutated bases underlined. The *lys2CT_1265_GA* mutation was similarly created by delitto perfetto. The genotypes of strains used in these experiments are given in Table S2. All gene deletions were created by one-step disruption with PCR generated fragments. In general gene deletions were made from a PCR fragment generated from the collection of yeast gene deletions [56]. The kanMX4 resistance marker was changed to hphMX4 or natMX4 by transformation with a fragment from pAG32 or pAG25, respectively [57]. For the *msh6Δ::loxP* deletion, the PCR fragment was from a strain in which *MSH6* had been disrupted by a loxP-kanMX-loxP fragment that was subsequently excised by Cre expression [58].

### Yeast transformation

Transformation was a modification of the method used previously [43], [59]. An overnight culture of a strain was diluted 1∶50 in YPAD [60], incubated with shaking at 30° to an OD_600_ of 1.3–1.5, washed twice with cold H_2_O, and once with cold 1 M sorbitol. After the final centrifugation, all solution was removed from the cells and a volume of cold 1 M sorbitol equal to that of the cell pellet added to resuspend the cells. For a typical transformation, 200 pmol of a Trp oligo and 200 pmol of LYS2TCARev40 (used to revert the *lys2CT_1265_GA* mutation) was added to 200 µl of this cell suspension in a 2-mm gap electroporation cuvette, and the mixture electroporated at 1.55 kV, 200 Ω, and 25 µF (BTX Harvard Apparatus ECM 630). Immediately after electroporation, the cell suspension was added to a volume of YPAD equal to that of the initial culture, and the cells incubated at 30° with shaking for 2 h. Cells were then centrifuged, washed with H_2_O, and plated on synthetic dextrose (SD) medium lacking either tryptophan or lysine [60] to select transformants. The number of Lys+ transformants served as a useful guide that a particular transformation experiment had worked, but was not correlated well enough with the number of Trp+ transformants to be used as an internal control (results not shown).

### Colony PCR and revertant analysis

Individual Trp+ revertants were picked into 200 µl SD-Trp medium in 96-well deep well plates, grown overnight at 30° with shaking, a small aliquot of each transferred to fresh SD-Trp medium with a Boekel Microplate Replicator and grown overnight, and finally transferred with the replicator to another deep well plate for overnight growth in 300 µl YPAD. Cells were then transferred with the replicator to a PCR microplate containing 15 µl per well of 2 mg/mL Zymolyase 20T (USBiological) in 0.1 M Phosphate Buffer pH 7.4 and incubated at 37° for 30 min and 95° for 10 min. After incubation, 85 µl H_2_O was added to each well. PCR was performed using 5 µl of the lysate in a total volume of 50 µl of the recommended buffer with 0.3 µM trpseq2 and trpseq8 primers [43] and 0.5 µl Takara e2TAK DNA polymerase for 30 cycles. For restriction digestion, 5 µl of the PCR reaction was incubated with 2 units of either *Bfa*I or *Sph*I (New England Biolabs) in the recommended buffer in a total volume of 15 µl at 37° overnight and analyzed by gel electrophoresis.

### Data analysis

The number of revertant colonies analyzed for each strain and oligo are given in Table S1. For each combination, usually 48 colonies were analyzed; in some instances experiments were repeated multiple times. For experiments repeated three or more times, means and standard deviations are shown in the figures. For comparison of results between strains, all data from a given strain and oligo were combined. Statistical calculations were performed using the VassarStats website (http://www.vassarstats.net/). In most cases, the number of samples was large enough for use of a chi-square test; in the remainder of cases, a Fisher's exact test was used. P values are given for each comparison in the relevant figure.

## Supporting Information

Figure S1 Template switching induced by 8-oxoG. This figure is similar to Figure 3A, except in strains with the F orientation. Template switching was determined as those transformants with Oligo G or Oligo GO that did not contain the *Sph*I restriction site introduced by the oligo. More than 40 Trp+ revertants were assayed for the presence of an *Sph*I site in each strain of the indicated genotype. The 8-oxoG lesion is replicated in the second round; thus replication is on the lagging strand in these strains. The error bars and horizontal bars are as in Figure 3. For **a**: P<0.0001.
(TIF)

Figure S2 Accuracy of 8-oxoG replication. This figure is similar to Figure 4A, except in strains of the F orientation transformed with Oligo GO. Trp+ revertants containing an *Sph*I site were assayed for the presence of a *Bfa*I site resulting from insertion of an A opposite 8-oxoG in strains of the indicated genotype transformed with an 8-oxoG-containing oligo; those transformants lacking the *Bfa*I site had a C inserted opposite the 8-oxoG. In most cases, more than 40 *Sph*I containing revertants were assayed. Note that the 8-oxoG lesion is replicated in the second round; thus replication is on the lagging strand in these strains. The error bars and horizontal bars are as in Figure 3. For **a**: P<0.0001; **b**: P = 0.02; **c**: P = 0.03.
(TIF)

Table S1 Number of transformants in the indicated category analyzed for each genotype.
(DOCX)

Table S2 Genotypes of strains.
(DOCX)
