# Supplementary figures and images for: In Vivo Bypass of 8-oxodG
Source: PLoS Genet. 2013 Aug 1;9(8):e1003682. doi: 10.1371/journal.pgen.1003682 (PMC3731214; doi:10.1371/journal.pgen.1003682)

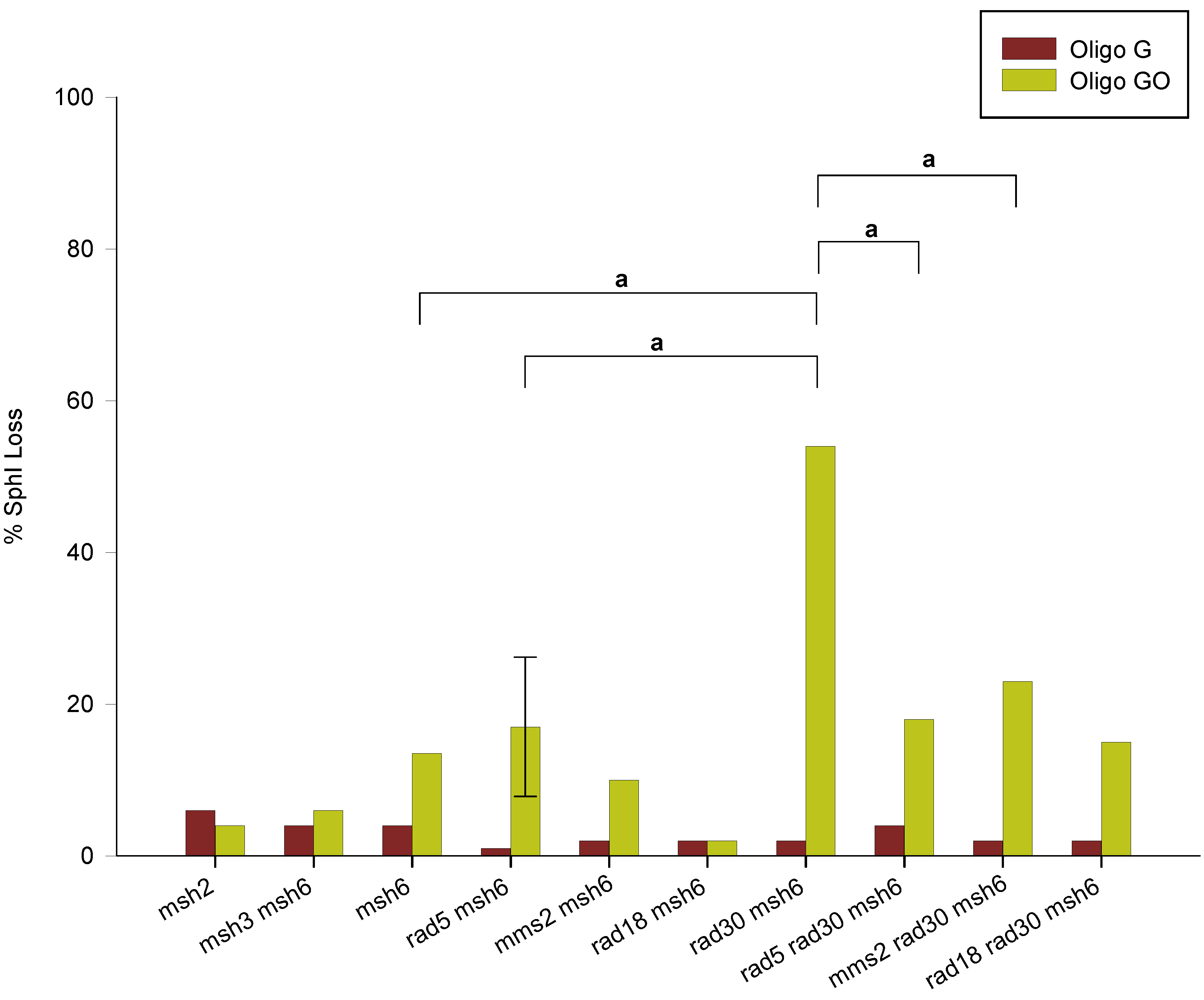

Supplement: Figure S1 — Template switching induced by 8-oxoG. This figure is similar to Figure 3A, except in strains with the F orientation. Template switching was determined as those transformants with Oligo G or Oligo GO that did not contain the SphI restriction site introduced by the oligo. More than 40 Trp+ revertants were assayed for the presence of an SphI site in each strain of the indicated genotype. The 8-oxoG lesion is replicated in the second round; thus replication is on the lagging strand in these strains. The error bars and horizontal bars are as in Figure 3. For a: P<0.0001. (TIF) [file pgen.1003682.s001.tif]

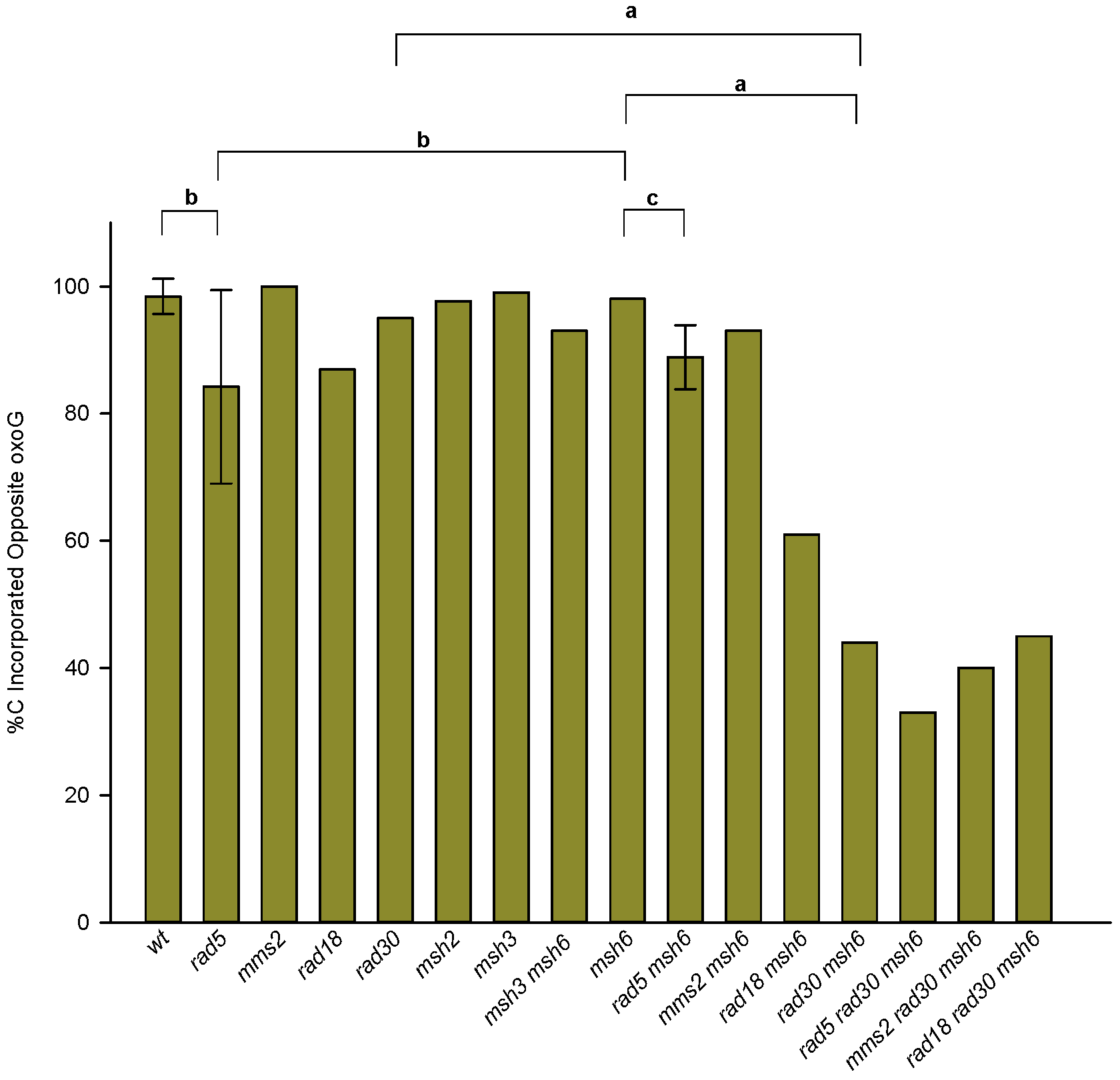

Supplement: Figure S2 — Accuracy of 8-oxoG replication. This figure is similar to Figure 4A, except in strains of the F orientation transformed with Oligo GO. Trp+ revertants containing an SphI site were assayed for the presence of a BfaI site resulting from insertion of an A opposite 8-oxoG in strains of the indicated genotype transformed with an 8-oxoG-containing oligo; those transformants lacking the BfaI site had a C inserted opposite the 8-oxoG. In most cases, more than 40 SphI containing revertants were assayed. Note that the 8-oxoG lesion is replicated in the second round; thus replication is on the lagging strand in these strains. The error bars and horizontal bars are as in Figure 3. For a: P<0.0001; b: P = 0.02; c: P = 0.03. (TIF) [file pgen.1003682.s002.tif]
