# Supplementary material for: In Vivo Bypass of 8-oxodG
Source: PLoS Genet. 2013 Aug 1;9(8):e1003682. doi: 10.1371/journal.pgen.1003682 (PMC3731214; doi:10.1371/journal.pgen.1003682)
Supplement: Table S1 — Number of transformants in the indicated category analyzed for each genotype. (DOCX) [file pgen.1003682.s003.docx]

| **Table S1**. Number of transformants in the indicated category analyzed for each genotype. | | | | | | | | | | | |  |  |  |  |  |
| --- | --- | --- | --- | --- | --- | --- | --- | --- | --- | --- | --- | --- | --- | --- | --- | --- |
|  | **F** | | | | | |  | **R** | | | | | | | |  |
|  | Trp+ | Trp+ | *Sph*I+ | | *Bfa*I+ | | % C | Trp+ | Trp+ | *Sph*I+ | *Bfa*I+ | | % C | | | |
| **Genotype** | **Oligo G** | **Oligo GO** | | | | |  | **Oligo G** | **Oligo GO** | | | | | |  | |
| wt | 70(2) | 132(3) | | 63 | | 1 | 98 | 48 | 143(3) | 96 | 3 | | | 97 | | |
| *rad5* | 60(2) | 159(4) | | 88 | | 12 | 86 | 60(2) | 153(4) | 103 | 12 | | | 88 | | |
| *rad18* | 147(2) | 79(2) | | 46 | | 6 | 87 | 147(2) | 96(2) | 59 | 2 | | | 97 | | |
| *rad30* | 60(2) | 124(2) | | 85 | | 4 | 95 | 60(2) | 154(2) | 114 | 6 | | | 95 | | |
| *msh3* | 95(2) | 190(4) | | 86 | | 1 | 99 | 46 | 48 | 33 | 1 | | | 97 | | |
| *msh2* | 47 | 46 | | 44 | | 1 | 98 | 48 | 40 | 39 | 3 | | | 92 | | |
| *msh6* | 48 | 95(2) | | 82 | | 2 | 98 | 47 | 96(2) | 90 | 6 | | | 93 | | |
| *mms2* | 48 | 48 | | 22 | | 0 | 100 | 48 | 48 | 28 | 0 | | | 100 | | |
| *msh3 msh6* | 47 | 47 | | 44 | | 3 | 93 | 48 | 47 | 45 | 6 | | | 87 | | |
| *rad5 msh6* | 58(2) | 236(5) | | 196 | | 22 | 89 | 56(2) | 142(3) | 136 | 27 | | | 80 | | |
| *rad18 msh6* | 47 | 47 | | 46 | | 18 | 61 | 48 | 93 (2) | 90 | 35 | | | 61 | | |
| *rad30 msh6* | 58(2) | 98(2) | | 45 | | 25 | 44 | 58(2) | 253(3) | 127 | 52 ^a^ | | | 36 | | |
| *mms2 msh6* | 48 | 48 | | 43 | | 3 | 93 | 48 | 47 | 44 | 6 | | | 86 | | |
| *rad5 rad30 msh6* | 47 | 95(2) | | 78 | | 52 | 33 | 48 | 48 | 48 | 27 | | | 44 | | |
| *rad18 rad30 msh6* | 48 | 47 | | 40 | | 22 | 45 | 47 | 46 | 45 | 29 | | | 36 | | |
| *mms2 rad30 msh6* | 48 | 95(2) | | 73 | | 44 | 40 | 43 | 48 | 42 | 29 | | | 31 | | |
|  | **Oligo UG** | **Oligo UGO** | | | | |  | **Oligo UG** | **Oligo UGO** | | | | |  | | |
| wt | 22 | 66(2) | | 63 | |  |  | 23 | 111(3) | 105 |  | | |  | | |
| *rad30* | 46 | 74(2) | | 66 | | 2 | 97 | 45 | 48 | 40 | 3 | | | 93 | | |
| *msh6* |  | 48 | | 48 | | 2 | 96 |  | 45 | 45 | 4 | | | 91 | | |
| *rad18 msh6* | 48 | 46 | | 45 | | 26 | 42 | 47 | 45 | 44 | 23 | | | 48 | | |
| *rad30 msh6* | 48 | 93(2) | | 66 | | 16^b^ | 41 | 48 | 141(3) | 93 | 14^c^ | | | 53 | | |
| *rad30 msh2* |  |  | |  | |  |  |  | 44 | 28 | 17 | | | 39 | | |
| *rad5 rad30 msh6* | 48 | 94(2) | | 91 | | 32^d^ | 29 | 48 | 89(2) | 86 | 31^e^ | | | 26 | | |

The table above gives for each orientation (F and R) and genotype the number of Trp+ revertants screened. In addition for Oligo GO and Oligo UGO, the number of those Trp+ revertants that were *Sph*I+ and the number of *Sph*I+ revertants that were *Bfa*I+ are given. The number in parentheses is the number of independent experiments. % C is the accuracy, as measured by the number of *Bfa*I- revertants divided by the number of *Sph*I+ revertants analyzed.

^a^Only 81 *Sph*I colonies were analyzed.

^b^Only 27 *Sph*I colonies were analyzed.

^c^Only 30 *SphI* colonies were analyzed.

^d^Only 45 *SphI* colonies were analyzed.

^e^Only 42 *SphI* colonies were analyzed.
