# Supplementary material for: In Vivo Bypass of 8-oxodG
Source: PLoS Genet. 2013 Aug 1;9(8):e1003682. doi: 10.1371/journal.pgen.1003682 (PMC3731214; doi:10.1371/journal.pgen.1003682)
Supplement: Table S2 — Genotypes of strains. (DOCX) [file pgen.1003682.s004.docx]

| **Table S2.** Genotypes of strains. | | |
| --- | --- | --- |
| **Strain Number** | ***TRP5* Orientation** | **Relevant Genotype** |
| GCY2196 | F | *MATα his3Δ200 ura3-52 leu2Δ1 trp5G148Cm lys2CT_1265_GA* |
| GCY2297 | R | *MATα his3Δ200 ura3-52 leu2Δ1 trp5G148Cm lys2CT_1265_GA* |
| GCY2335 | F | GCY2196 *msh6Δ::hygMX* |
| GCY2336 | R | GCY2297 *msh6Δ::hygMX* |
| GCY2414 | F | GCY2196 *msh2Δ::hphMX4* |
| GCY2408 | R | GCY2297 *msh2Δ::hygMX* |
| GCY2431 | F | GCY2196 *rad30Δ::kanMX* |
| GCY2432 | R | GCY2297 *rad30Δ::kanMX* |
| GCY2433 | F | GCY2196 *msh6Δ::hygMX* *rad30Δ::kanMX* |
| GCY2434 | R | GCY2297 *msh6Δ::hygMX* *rad30Δ::kanMX* |
| GCY2458 | F | GCY2196 *msh3Δ::kanMX* |
| GCY2459 | R | GCY2297 *msh3Δ::kanMX* |
| GCY2475 | F | GCY2196 *rad5Δ::kanMX* |
| GCY2476 | R | GCY2297 *rad5Δ::kanMX* |
| GCY2477 | F | GCY2196 *rad18Δ::kanMX* |
| GCY2478 | R | GCY2297 *rad18Δ::kanMX* |
| GCY2492 | F | GCY2196 *msh6Δ::hygMX* *rad18Δ::kanMX* |
| GCY2493 | R | GCY2297 *msh6Δ::hygMX* *rad18Δ::kanMX* |
| GCY2516 | F | GCY2196 *msh6Δ::hygMX* *rad18Δ::kanMX* *rad30Δ::natMX* |
| GCY2517 | R | GCY2297 *msh6Δ::hygMX* *rad18Δ::kanMX* *rad30Δ::natMX* |
| GCY2520 | F | GCY2196 *msh6Δ::hygMX* *rad5Δ::natMX* *rad30Δ::kanMX* |
| GCY2521 | R | GCY2297 *msh6Δ::hygMX* *rad5Δ::natMX* *rad30Δ::kanMX* |
| GCY2525 | F | GCY2196 *msh6Δ::hygMX* *rad5Δ::kanMX* |
| GCY2537 | R | GCY2297 *msh6Δ::hygMX* *rad5Δ::natMX* |
| GCY2588 | F | GCY2196 *msh3Δ::kanMX* *msh6Δ::hygMX* |
| GCY2589 | R | GCY2297 *msh3Δ::kanMX* *msh6Δ::hygMX* |
| GCY2600 | F | GCY2196 *mms2Δ::HIS3MX* |
| GCY2601 | R | GCY2297 *mms2Δ::HIS3MX* |
| GCY2602 | F | GCY2196 *msh6Δ::hygMX* *mms2Δ::HIS3MX* |
| GCY2603 | R | GCY2297 *msh6Δ::hygMX* *mms2Δ::HIS3MX* |
| GCY2604 | F | GCY2196 *msh6Δ::hygMX* *rad30Δ::kanMX* *mms2Δ::HIS3MX* |
| GCY2605 | R | GCY2297 *msh6Δ::hygMX* *rad30Δ::kanMX* *mms2Δ::HIS3MX* |
| GCY2753 | R | GCY2297 *msh2Δ::hygMX* *rad30Δ::natMX* |
